# Cloud-based services for electronic civil registration and vital statistics systems

**DOI:** 10.1186/s41043-019-0181-5

**Published:** 2019-10-18

**Authors:** Brett McDowall, Samuel Mills

**Affiliations:** 1 Object Consulting Pty Ltd, Level 25 Northpoint, 100 Miller Street, North Sydney, NSW 2060 Australia; 2 World Bank Group, 1818 H Street NW, Washington DC, 20433 USA

**Keywords:** Cloud-based service, Data center, Civil registration and vital statistics

## Abstract

This paper examines the hosting options for electronic civil registration and vital statistics (CRVS) systems, particularly the use of data centers versus cloud-based solutions. A data center is a facility that houses computer systems and associated hardware and software components, such as network and storage systems, power supplies, environment controls, and security devices. An alternative to using a data center is cloud-based hosting, which is a virtual data center hosted by a public cloud provider. The cloud is used on a pay-as-you-go basis and does not require purchasing and maintaining of hardware for data centers. It also provides more flexibility for continuous innovation in line with evolving information and communications technology.

## Main text

Most low- and middle-income countries are in the process of strengthening civil registration and vital statistics (CRVS) systems and moving from using paper records to electronic records. Some low-income countries such as Ethiopia, The Gambia, and Lao People’s Democratic Republic rely solely on paper records, while others such as Liberia and Rwanda have limited CRVS electronic databases. These countries, even with their CRVS systems at different stages, are all striving to establish a secure and effective electronic CRVS system. Choosing an appropriate hosting option is one of the key elements that require governments to make critical choices for establishing a cost-effective and secure electronic CRVS system.

This paper compares the use of data centers versus cloud-based solutions for electronic CRVS systems. While District Health Information Software 2 (DHIS2), the most commonly used health information system in low- and middle-income countries, is often cloud-based [1], to our knowledge, there has not been any previous publication describing the merits and demerits of different hosting options for electronic CRVS systems.

**Fig. 1:**
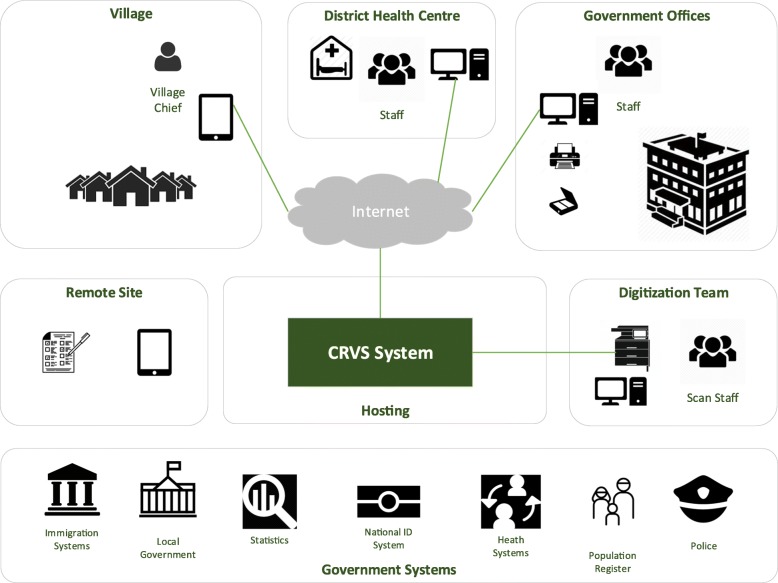
Conceptual architecture of a typical CRVS system

A typical CRVS system solution architecture has the following elements (Fig. 1):

I. User devices—such as laptops, desktops, tablets, and mobile phones that system users use in government offices, health centers, villages, homes, and in the field, including scanners for digitizing existing records and storing copies of paper documents that have been submitted.
II. Connectivity—connection to the Internet, which allows communication between various parts of the system.
III. Software—for the CRVS system.
IV. Hosting—the deployed version of the system that runs on a set of servers connected to the Internet via network devices. These servers and networks could be virtual in the cloud or could be located in a government data center.
V. Digitization—the process of scanning and storing existing paper records in the system with sufficient index information (such as name and date) to allow them to be found when needed.
VI. Government Systems—the integration with a range of systems that represent the eGovernment ecosystem.

**Fig. 2:**
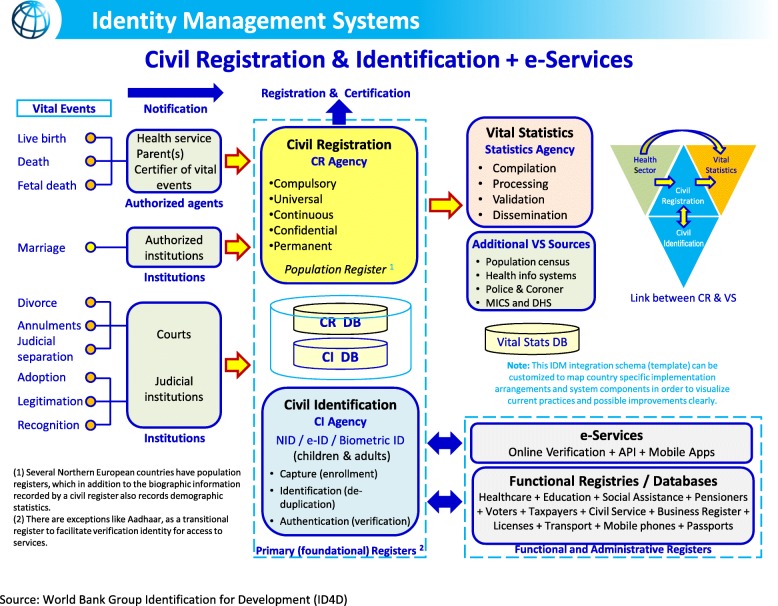
Integration of civil registration, vital statistics, and identity management systems

Figure 2 depicts a holistic integration of civil registration, vital statistics, and identity management systems.

## Data center

Most policymakers are more familiar with data centers than cloud-based solutions. A CRVS system running in a data center could use physical servers and storage or it could use their virtualized counterpart “cloud”. A data center is a facility that houses computer systems and associated components, such as network and storage systems, and includes power supplies, environment controls, data connections, and security devices. Government agencies have typically run their own data centers or data centers of different agencies have been consolidated into interconnected data centers thereby creating “whole of government data centers”.

Uptime Institute, a data center research and professional-services organization which has certified over 1000 leading data center facilities worldwide for design, construction, management, and operations, has a four-tier rating system [2] for data centers, as follows:

Tier I (basic capacity): Tier I infrastructure includes a dedicated space for information technology (IT) systems; an uninterruptible power supply (UPS) to filter power fluctuations and outages; dedicated cooling equipment that runs 24/7; and a backup generator to power IT equipment during extended power outages.

Tier II (redundant capacity components): Tier II facilities include redundant power and cooling components to allow for maintenance opportunities and an increased margin of safety against IT process disruptions resulting from equipment failures. The redundant components include UPS modules, chillers, pumps, and engine generators.

Tier III (concurrently maintainable): A Tier III data center can maintain and replace equipment without shutting down. A redundant delivery path for power and cooling is added to the redundancy incorporated by Tier II so that every component needed to support the IT environment can be shut down and maintained without impact on the overall IT operation.

Tier IV (fault tolerance): Tier IV adds the concept of fault tolerance to the site infrastructure, so when individual equipment failures or distribution interruptions occur, the data center’s IT operation is not affected.

These descriptions above highlight that a reliable and trusted data center is more than simply a server room that holds racks of servers. An electronic CRVS system is expected to run on a Tier III or Tier IV data center, which implies considerable complexity and commitment to ongoing operational support and investment in upgrades. Data centers typically become obsolete after 7 years and require upgrades every 2–3 years. To build a data center for eCRVS, a total cost of ownership model of analysis, which takes into consideration the initial capital investment plus maintenance and operational costs, is recommended [3]. Given the huge costs involved, a large data center that serves several government agencies is more cost-effective than an isolated data center that serves just the CRVS system [4].

## Data center versus cloud-based solutions

An alternative to hosting the CRVS system by using a data center is using cloud-based hosting which is a virtual data center hosted by a public cloud provider where data can be encrypted so the provider cannot see the data. The National Institute of Standards and Technology defines cloud computing as “a model for enabling ubiquitous, convenient, on-demand network access to a shared pool of configurable computing resources (such as, networks, servers, storage, applications, and services) that can be rapidly provisioned and released with minimal management effort or service provider interaction” [5].

Cloud computing could be public, private, or a hybrid. A public cloud is owned by a private entity and the computing resources such as servers are delivered via the Internet for a fee. A private cloud is owned by a government or an organization that leverages the resources of data centers in different locations of its own use, while hybrid cloud computing refers to a combination of the public and private clouds. There are a number of public cloud service providers with different offerings (storage, database, and network) and different pricing. Notable providers are Amazon, Microsoft, Google, HP, and IBM. Examples of commonly used cloud-based applications are Microsoft Office Suite, Dropbox, Gmail, and WebEx.

Data centers need scale to be cost effective, and the ultimate cost-effective data centers are those offered by the public cloud providers. The benefits of the cloud are that electronic CRVS systems can be started-up quickly without the need to build a data center; the price is low with pay-as-you-go plans; and it also offers flexibility, high levels of security, and the ability to support innovation.

The price of using a public cloud for CRVS systems could range from about US$0.02 per hour to about US$0.10 per hour for a single lower-end server (that is, between US$180 per year and US$880 per year), covering all costs, including purchase, setup, installation, networks, power, and cooling [6]. It also comes with an operating system and often some storage. On the other hand, using a data center will require buying a server, installing it, configuring it, powering it, and eventually upgrading it. For instance, a low-end dual central processing unit server with 16GB Random Access Memory, 2x256GByte Solid State Disk Drives, and a 2x1TByte hard disk drive will cost about US$5000, but in addition, it will require floor space, rack space, power supply, electricity, cooling, and monitoring. Cloud-based systems often have per-user licensing models, with different tiers where the price goes up with the need for more services. It is imperative to ensure that a copy of the CRVS data is securely sent to a government data center in a reliable manner on a regular basis—no less than daily, but ideally hourly or even in delayed real time. This requires a simple server and reliable storage which would cost about US$1000.

Although using a public cloud is cheaper than building and maintaining a data center, there are concerns or misconceptions about cloud-based services, such as issues regarding data sovereignty and security. Table 1 presents some of these concerns and responses to them. If a government decides to begin using cloud services, it should ensure that it has the appropriate legislation in place to allow data to reside offshore or that there is no legislation that explicitly precludes the use of cloud storage. The DHIS2 implementer guide examined three options for server hosting: (a) server in the Ministry of Health (e.g., Bangladesh), (b) sever in a government data center (e.g., Bhutan), and (c) cloud-based (e.g., Liberia), and indicated that cloud-based hosting is the most cost-effective option [7]. It is imperative for Governments to define the standards that any cloud system needs to meet covering data sovereignty, ownership, security, availability, and performance. For instance, the New Zealand government adopted a “Cabinet’s Cloud First policy which requires agencies to adopt cloud services in preference to traditional IT systems because they are more cost effective, agile, are generally more secure, and provide greater choice” [8]. However, the New Zealand Government requires each agency to first assess the risk and take mitigation measures [9]. Similarly, the Australian Government also has in place guidelines for cloud computing cybersecurity [10].

**Table 1:** Risks, concerns, and responses regarding cloud-based services

Risk or concern	Response
We will be losing control; we will not know what we have and cannot guarantee anything.	Cloud providers provide extensive tools and consoles, so it is easy to see what is happening at all times, which can be better than using one’s own data center.
We will be totally changing the skills we need; detailed technical work will no longer be needed, and the focus will be more on the architecture.	This is a shift, and it means that you do not need to worry about many of the technical details, but the client or the vendor must make sure the architecture and design are right. Because hardware is now more like software, it is easier to change everything.
Surely the security cannot be as good as if we do it ourselves; the system is running in a big data center somewhere else.	Cloud providers have excellent security at all levels and have the volume to ensure that they do it right. They use automated tools and see and handle many different attacks and scenarios every day.
Once we go into this, we cannot come back; there will be serious vendor lock-in.	It is possible to not use cloud provider-specific features, so that it is easy to move between vendors. You could commission a proof of concept of the system running on a different cloud if desired.
It looks hard to get everything right upfront; you really need to design everything properly before it will work.	You would need to do the same with a data center, but working in the virtual world provides more flexibility, and the way the cloud works means that everything needs to be set up explicitly, which is a good thing.
This is a major change to how we do things, and it will cause more disruption than it is worth.	The cloud and associated support partners take a lot of the server-related work away and allow you to focus on core CRVS challenges that make a difference. There can be a short adjustment period, and some people may need to be reassigned. It will no longer be necessary to maintain large amounts of hardware, or large groups of support and security specialists for the long term, and this change can save a significant amount of money and human resources for the country.
The cloud can fail, and then we will be totally out of action.	That is true, but it does not happen often, and the period of disruption is normally short. Given that a cloud provider uses multiple centers, there is no single point of failure, although in an extreme case, the system could be unavailable for up to a few hours—which a CRVS system can tolerate.
We are just not convinced it is going to end up being cheaper; servers do not cost that much these days.	They do not cost much, but cloud vendors buy their own specialized servers by the tens of thousands, so they can buy them less expensively. They can ensure that they have high utilization and can amortize the housing, cooling, power, management, and support costs.
There must be hidden costs or pricing bands that will catch up with us at some point.	The cloud is a mature marketplace in 2019, and there is enough competition that providers will be transparent with the pricing. All costs are stated on their websites, and they provide calculators to help; one needs to check not just on computing and storage costs, but also things such as data flows, domain name servers, and directories. The biggest challenge with costs is managing accounts and knowing what is happening. It is easy to have many environments—some overprovisioned—and to leave them running all the time.
We need to be flexible; right now, we can do anything we like with the hardware and the networks.	Cloud providers are always developing new services to allow clients to do exactly what they need. It is a big market, and they have millions of customers, so things are evolving rapidly.
Customer support could be slow, because the providers are large companies that handle many customers.	There have been concerns raised about customer support, but providers all have partner programs for software developers and support organizations that they work with, and there are specific support channels that help. In most cases, you do not need support because the systems and tools provided do what they say they do.
Performance in the cloud could be poor.	In the past, there have been issues with legacy systems not performing well in the cloud. But the services offered by cloud providers have been improved to allow a broader range of legacy systems to be run unchanged in the cloud. In addition, the increasing number of modern systems which have been built to run in distributed dynamically scalable environments means that in practice it is much less of an issue.

It is noted that some countries legally require all government systems to be locally hosted, while others require it for only specific systems, for example, requiring health, business, or payment records to be stored locally. The key point is to ensure that the relative merits and demerits of cloud versus local hosting are clearly understood. The CRVS Digitization Guidebook includes a discussion of the pros and cons of different platform types and hosting options [11].

The cloud is very much the way of the future for hosting modern government systems. However, at the same time, steps should be taken to ensure that only authorized users, including a tightly controlled set of administrators working for support partners and cloud administrators themselves, can access sensitive data. Encrypting databases is a simple technique that makes it impossible for anyone to simply take a copy of the data. Care must be taken with system logs and audit trails to ensure that they do not contain sensitive information as their access is controlled as a part of the CRVS system itself, rather than through the cloud infrastructure.

## Conclusion

The cloud is used on a pay-as-you-go basis, so there is no need to buy unnecessary services or hardware, and processing power and storage volumes can be changed at will, which reduces waste and makes planning much simpler. It also provides considerable flexibility and removes one of the major barriers to innovation in information and communication technology, namely the need for purchasing and provisioning hardware for new services which may or may not be successful.

A cloud provider’s full-time job is to monitor security and manage all possible threats. This is more efficient than doing it in-house since the public cloud service provider can spread the cost across many thousands of customers. With the cloud, the CRVS data is only available for authorized use, and the potential for an administrator or an IT staff being able to do almost anything and viewing everything is minimized, which also makes data theft less common.

Business continuity is always important. Cloud-based services provide quick data recovery for all kinds of emergency scenarios from natural disasters to power outages. Cloud providers utilize multiple data centers which are connected via multiple high-speed links, so failure can be handled without disruption.

The cloud allows adoption of new platforms or changes to the architecture of the systems. Without the constraints of fixed hardware and with the ability to pay-as-you-go, it is easy to create new systems or change the cloud providers to use new applications or services.

For governments to make informed decisions about hosting of CRVS systems, they need to consider the legislation of their country, the capability of their local hosting operations, the business and technical requirements for a CRVS system, data sovereignty policies relating to other countries, and the contracts and services on offer from CRVS vendors along with the longer-term trends in the information and communications technology sector.

## Data Availability

Not applicable

## Abbreviations

CRVS: Civil registration and vital statistics
DHIS2: District Health Information Software 2
IT: Information technology
UPS: Uninterruptable power supply
